# Comparison of the Current Diagnostic Criterion of HbA1c with Fasting and 2-Hour Plasma Glucose Concentration

**DOI:** 10.1155/2016/6195494

**Published:** 2016-08-11

**Authors:** Rudruidee Karnchanasorn, Jean Huang, Horng-Yih Ou, Wei Feng, Lee-Ming Chuang, Ken C. Chiu, Raynald Samoa

**Affiliations:** ^1^Division of Endocrinology, Department of Medicine, University of Kansas Medical Center, Kansas City, KS 66160, USA; ^2^Department of Clinical Diabetes, Endocrinology, and Metabolism, City of Hope National Medical Center, Duarte, CA 91010, USA; ^3^Division of Endocrinology, Metabolism and Nutrition, Department of Internal Medicine, Harbor-UCLA Medical Center, Torrance, CA 90502, USA; ^4^Division of Endocrinology and Metabolism, Department of Internal Medicine, National Cheng Kung University Hospital, College of Medicine, National Cheng Kung University, Tainan, Taiwan; ^5^Department of Internal Medicine, National Taiwan University Hospital, Taipei, Taiwan; ^6^Graduate Institute of Preventive Medicine, School of Public Health, National Taiwan University, Taipei, Taiwan

## Abstract

To determine the effectiveness of hemoglobin A1c (HbA1c) ≥ 6.5% in diagnosing diabetes compared to fasting plasma glucose (FPG) ≥ 126 mg/dL and 2-hour plasma glucose (2hPG) ≥ 200 mg/dL in a previously undiagnosed diabetic cohort, we included 5,764 adult subjects without established diabetes for whom HbA1c, FPG, 2hPG, and BMI measurements were collected. Compared to the FPG criterion, the sensitivity of HbA1c ≥ 6.5% was only 43.3% (106 subjects). Compared to the 2hPG criterion, the sensitivity of HbA1c ≥ 6.5% was only 28.1% (110 subjects). Patients who were diabetic using 2hPG criterion but had HbA1c < 6.5% were more likely to be older (64 ± 15 versus 60 ± 15 years old, *P* = 0.01, mean ± STD), female (53.2% versus 38.2%, *P* = 0.008), leaner (29.7 ± 6.1 versus 33.0 ± 6.6 kg/m^2^, *P* = 0.000005), and less likely to be current smokers (18.1% versus 29.1%, *P* = 0.02) as compared to those with HbA1c ≥ 6.5%. The diagnostic agreement in the clinical setting revealed the current HbA1c ≥ 6.5% is less likely to detect diabetes than those defined by FPG and 2hPG. HbA1c ≥ 6.5% detects less than 50% of diabetic patients defined by FPG and less than 30% of diabetic patients defined by 2hPG. When the diagnosis of diabetes is in doubt by HbA1c, FPG and/or 2hPG should be obtained.

## 1. Introduction

Diabetes has reached epidemic proportions in the US and worldwide [1]. The estimated total economic cost of diagnosed diabetes in 2012 was $245 billion in the US alone [2]. Diabetes and its complications are serious and potentially life-threatening, but the disease process can be halted or slowed by effective treatment [3]. In 2014, according to the Center for Disease Control and Prevention, 25.8 million people in the United States have diabetes and 1.9 million people aged 20 years or older were newly diagnosed in 2010 alone based on either fasting plasma glucose (FPG) or hemoglobin A1c (HbA1c) criterion [4]. Seven million people with diabetes are undiagnosed, and a large percentage of newly diagnosed individuals already have complications at the time of diagnosis. Because most patients with early diabetes are asymptomatic, effective screening tests are crucial, and early identification of diabetes and initiation of appropriate treatment are vital to patients' health. Evidence suggests that early diagnosis and proper treatment of type 2 diabetes confer health benefits, whereas aggressive control of blood glucose, blood pressure, and cholesterol after diagnosis of type 2 diabetes may be less important than early screening [5]. Additionally, delaying screening and treatment for type 2 diabetes may hasten and/or increase the risk for developing cardiovascular diseases. Thus, reliable screening and diagnostic methods are necessary to identify individuals at risk. To achieve this goal, a simplified diagnostic test has been proposed by the measurement of HbA1c by various organizations.

HbA1c is produced by a nonenzymatic reaction that occurs between glucose and hemoglobin, which was first characterized in 1968 [6]. Elevated HbA1c in diabetic patients was first reported by Rahbar et al. in 1969 [7]. As plasma glucose increases, the fraction of HbA1c increases in a predictable way. This serves as a surrogate marker for average blood glucose levels over the previous months prior to the measurement. Subsequently, the clinical application of HbA1c in monitoring glycemic control in diabetic patients was demonstrated in 1976 [8]. Since then, the measurement of HbA1c has become a standard in the care of patients with diabetes and for monitoring glycemic control over a 3-month period. Aggressive improvement in glycemic control, as demonstrated by a reduction in HbA1c, reduced the rate of diabetic complications and improved quality of life [9]. However, due to lack of standardization of the HbA1c assay, it was not until 2009 that HbA1c was incorporated as a diagnostic tool for diabetes [10]. This was mainly achieved through the effort of the National Glycohemoglobin Standardization Program (NGSP) [11]. In 2010, the American Diabetes Association included HbA1c ≥ 6.5% (48 mmol/mol) as a diagnostic criterion based on its correlation with retinopathy [10]. Since then, only one longitudinal study has validated the inflection point of HbA1c ≥ 6.5% (48 mmol/mol) for increased incidence of retinopathy [12], and other longitudinal studies have suggested that the inflection point for retinopathy may not be at HbA1c of 6.5% (48 mmol/mol) [12–16]. For example, McCance et al. studied the development of retinopathy and nephropathy in Pima Indians and found that the threshold for retinopathy based on HbA1c was at the 80th percentile, which corresponded to HbA1c ≥ 6.9% (52 mmol/mol) [14]. van Leiden et al. published the incidence of retinopathy in individuals from the Hoorn study, a population-based cohort study on glucose metabolism in Netherlands [16], and showed that the participants in the highest HbA1c group (HbA1c 5.8–13.1% or 40–120 mmol/mol) had 21.1% incidence of retinopathy, with an odds ratio of 3.95 (95% confidence interval: 1.19, 13.03). Because the range of HbA1c in this group was very wide, a clear cut-off point could not be determined. Selvin et al. studied the association between HbA1c and the risk of kidney disease and retinopathy in a community-based population during a median of 14 years of follow-up [15]. The study did not find an HbA1c threshold for microvascular outcomes before or after adjusting covariates. The DESIR study examined 700 subjects for development of retinopathy at 10-year follow-up and found that the positive predictive values for retinopathy increased sharply at HbA1c of 6.0% (42 mmol/mol) [13]. Based on these longitudinal studies, HbA1c ≥ 6.5% (48 mmol/mol) has not been validated as the inflection point at which the risk of retinopathy increases in the general population [17]. Thus, the current diagnostic cut-off for diabetes based on HbA1c is still in a quandary, and it is highly likely that the diagnostic criterion based on HbA1c will be revised in the future.

The diagnostic criteria of diabetes have been evolving over time. Diabetes was previously diagnosed by fasting plasma glucose (FPG) ≥ 140 mg/dL (7.8 mmol/L) or a 2-hour plasma glucose (2hPG) level ≥ 200 mg/dL (11.1 mmol/L), based on the criteria established by the National Diabetes Data Group in 1979 [18]. In 1997, the fasting glucose threshold was decreased to 126 mg/dL (7.0 mmol/L) [19], which was intended to reflect the discrepancy between 2hPG and FPG (many subjects have a 2hPG ≥ 200 mg/dL or 11.1 mmol/L and a FPG < 140 mg/dL or 7.8 mmol/L) and to simplify the diagnostic process (fasting blood test versus oral glucose tolerance test, OGTT). In 2009, HbA1c was defined as one of the diagnostic criteria for diabetes [10].

The National Health and Nutrition Examination Survey (NHANES) is a program designed to assess the health and nutritional status of adults and children in the United States. It has been conducting continuous surveys in different population groups across the country and surveys a variety of demographic, socioeconomic, dietary, and health groups. Based on the datasets from NHANES 2005–2010, we examined the effectiveness of using HbA1c in diagnosing diabetes compared to FPG and 2hPG in a previously undiagnosed diabetic cohort. As this was a cross-sectional study, we could not examine the impact of diagnostic criteria on the long-term diabetic complications. Instead, we investigated the agreement of the current diagnostic criteria for diabetes to facilitate the early diagnosis of diabetes in the present study.

## 2. Methods

### 2.1. Ethics Statement

The NHANES has been conducted by the National Center for Health Statistics of the Centers for Disease Control and Prevention in the United States since the 1960s. The purpose of this survey is to assess the health and nutrition status of a large representative sample in the United States and to provide vital and health statistics for the nation. The survey and data collection were approved by the NHANES Institutional Review Board (IRB) and documented consent was obtained from participants. Only deidentified data from the survey was used in this study, and its use is exempt from the federal regulations for the protection of human research participants as previously described [20].

### 2.2. Study Design and Study Sample

Detailed descriptions of the survey and the analytical methods of various assays have been updated regularly and are available at its website (http://www.cdc.gov/nchs/about/major/nhanes/datalink.htm#NHANESIII) and described previously [21].

### 2.3. Study Population

**Figure 1:**
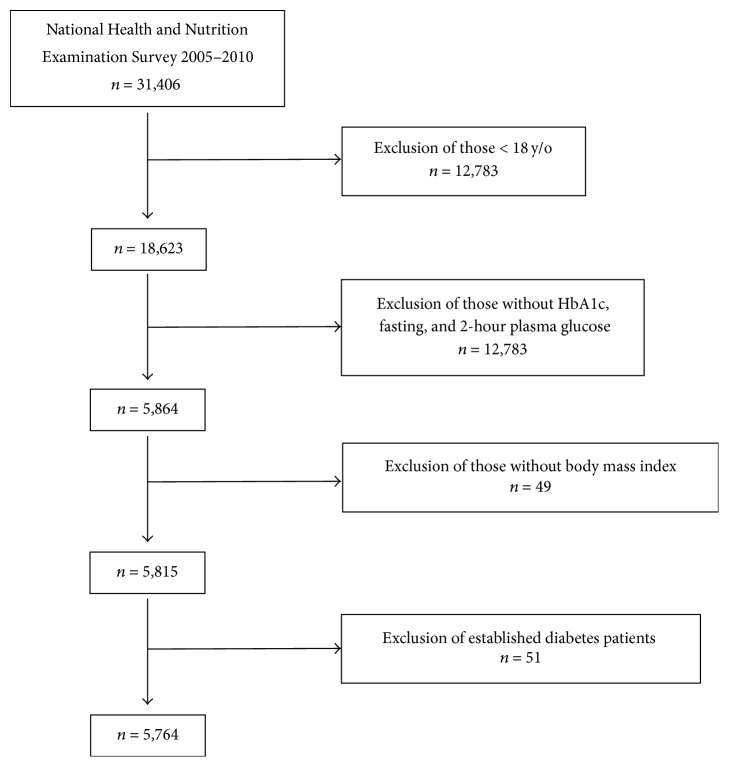
Sampling scheme.

NHANES is a population-based survey designed to be representative of the US civilian noninstitutionalized population. Starting in 2007, NHANES began oversampling all Hispanics. Previous survey periods (1999–2002 and 2003–06) oversampled Mexican Americans only and certain other groups (i.e., low income persons, adolescents, the elderly, and blacks). Databases from the NHANES from years 2005 through 2010 were evaluated for the study (*n* = 31,406). There were 5,815 subjects, 18 years old or older, who had a measured HbA1c, 2hPG, FPG, and body mass index (BMI). Those who had established diabetes based on the history of self-reported diabetes or by use of insulin and/or oral antidiabetic agents were excluded (*n* = 51). The present sample set consisted of 5,764 subjects without a prior diagnosis of diabetes (Figure 1).

### 2.4. Diagnostic Criteria of Diabetes

For this study, in accordance with the guidelines set forth by the American Diabetes Association, diabetes was defined as having a FPG ≥ 126 mg/dL (7.0 mmol/L), 2hPG ≥ 200 mg/dL (11.1 mmol/L), or HbA1c ≥ 6.5% (48 mmol/mol) [22].

### 2.5. Laboratory Methods

#### 2.5.1. HbA1c

HbA1c was measured using HPLC based assays. HbA1c measurements were determined using a Tosoh A1c 2.2 Plus Glycohemoglobin Analyzer during NHANES 2005-2006 and a Tosoh A1c G7 HPLC Glycohemoglobin Analyzer during NHANES 2007-2008 and 2009-2010. Although different HbA1c laboratory instruments and laboratories were used between 2005 and 2010, laboratory method crossover studies were conducted at the time of the laboratory instrument changes. Both laboratories analyzing NHANES HbA1c data from 1999 to 2010 were standardized by participating in the NGSP. A laboratory group from the NGSP system was consulted in February 2012 to review the NHANES laboratory and participant hemoglobin HbA1c data. The NGSP group concluded that both NHANES laboratories met NGSP criteria for bias and precision from 1999 to 2010. Thus, no crossover regression was made in the present study. The rereleased hemoglobin A1c data for 2007-2008 (GHB_E) and 2009-2010 (GHB_F) in March 2012 was used in this study.

#### 2.5.2. Plasma Glucose Concentration

Plasma glucose concentration was determined by a hexokinase method which is an endpoint enzymatic method using a sample blank correction. As OGTT was not performed between 1999 and 2004, we only included the 2005–2010 data in this analysis. Beginning in 2005, an OGTT was reintroduced to the laboratory protocol for NHANES. A fasting blood test was performed on all participants 12 years old and older; these participants were examined in the morning session, following a 9-hour fast. After the initial venipuncture, participants were asked to drink a calibrated dose (75 grams of glucose), and a second venipuncture was performed 2 hours (±15 minutes) later.

### 2.6. Statistical Analysis

Continuous data were expressed as mean ± standard deviation (STD), unless otherwise specified. Continuous differences were examined using a two-tail Student's *t*-test. Categorical differences were given in proportions and examined using a Chi-square test. *P* < 0.05 was considered statistically significant. Regression analysis was used to examine the relation of HbA1c with FPG and 2hPG. Cohen's kappa coefficient was calculated to assess the agreement between HbA1c and FPG and between HbA1c and 2hPG. The fitted receiver operating characteristic (ROC) curve was calculated using a web-based calculator, and the fitted ROC curve was plotted with 95% confidence interval (Eng J. ROC analysis: web-based calculator for ROC curves. Baltimore: Johns Hopkins University (updated September 11, 2007), available from http://www.jrocfit.org/. Access on October 20, 2013).

## 3. Results

### 3.1. Studied Population

The clinical characteristics of 5,764 subjects in this study are shown in Table 1. The study consisted of 1,117 Mexican Americans (19.4%), 519 other Hispanics (9.0%), 2,818 non-Hispanic Whites (48.9%), 1,060 non-Hispanic Blacks (18.4%), and 250 subjects of other racial/ethnic groups (4.3%). The cohort had a mean age of 46 years and a mean BMI of 28.4 kg/m^2^. Based on the established diagnostic criteria, 245 (4.3%) subjects were diabetic with FPG ≥ 126 mg/dL (7.0 mmol/L), 392 subjects (6.8%) were diabetic with 2hPG ≥ 11.1 mmol/L (200 mg/dL), and 146 (2.5%) subjects were diabetic with HbA1c ≥ 6.5% (48 mmol/mol).

### 3.2. Diabetes Based on Fasting Plasma Glucose versus HbA1c

Among 245 individuals that had FPG ≥ 126 mg/dL (7.0 mmol/L), 106 subjects (43.3%) had HbA1c ≥ 6.5% (48 mmol/mol), and 139 subjects (56.7%) had HbA1c <6.5% (48 mmol/mol) (Table 2). The sensitivity and specificity of HbA1c ≥ 6.5% in diagnosing diabetes mellitus based on FPG ≥ 126 mg/dL (7.0 mmol/L) were 43.3% and 99.3%, respectively. The sensitivity differed widely among ethnic groups (Mexican Americans 50.0%, other Hispanics 37.5%, non-Hispanic Whites 37.8%, non-Hispanic Blacks 51.4%, and others 80.0%). However, the specificity was greater than 98.0% in all ethnic groups. The positive predictive value and the negative predictive value were 72.6% and 97.5%, respectively.

Subjects that met the criteria for diagnosis of diabetes mellitus based on FPG ≥ 126 mg/dL (7.0 mmol/L) were further analyzed in two groups based on HbA1c (<6.5% versus ≥6.5%, 48 mmol/mol) (Table 3). There were no statistically significant differences between the two groups in regard to age, gender, blood pressure, current smoking, alcohol consumption, and family history of diabetes, except for HbA1c, FPG, and 2hPG. BMI approached significance (*P* = 0.08), because more subjects from the group HbA1c < 6.5% (48 mmol/mol) had BMI in nonobese range. Based on the difference in HbA1c, FPG, and 2hPG, the HbA1c criterion performed poorly in those with less elevated plasma glucose and less severe diabetes when compared to the FPG criterion.

### 3.3. Diabetes Based on 2-Hour Plasma Glucose versus HbA1c

Out of 5,764 subjects, only 392 patients (6.8%) had a diagnosis of diabetes using the 2hPG criterion (Table 4). Of those, only 110 (28.1%) had HbA1c ≥ 6.5% (48 mmol/mol). Thus, if only the HbA1c criterion was used in these cases to diagnose diabetes, about 72% of the patients would have had a missed diagnosis of diabetes. In reference to 2hPG, sensitivity for diagnosing diabetes using HbA1c was only 28.1% with an excellent specificity of 99.3%. The HbA1c criterion had a false positive rate of 0.7%, while the false negative rate was up to 71.9%. These results indicate that the current HbA1c criterion performed poorly as a sole indicator of the diagnosis of diabetes when compared to the current 2hPG criterion.

To further elucidate the likelihood that certain characteristics would predispose subjects to have HbA1c < 6.5% (48 mmol/mol) despite having 2hPG ≥ 200 mg/dL (11.1 mmol/L), we divided subjects into those with HbA1c < and those with HbA1c ≥ 6.5% (48 mmol/mol) and compared the two groups (Table 5). Patients with HbA1c < 6.5% (48 mmol/mol) were more likely to be older (64 ± 15 versus 60 ± 15 years old, *P* = 0.01), female (53.2% versus 38.2%, *P* = 0.008), and leaner by BMI (29.7 ± 6.1 versus 33.0 ± 6.6 kg/m^2^, *P* < 0.0001) and less likely to be current smokers (18.1% versus 29.1%, *P* = 0.02) as compared to those with HbA1c ≥ 6.5% (48 mmol/mol). Among 5 racial/ethnic groups, the current HbA1c criterion performed best in non-Hispanic blacks by successfully identifying 51.2% of subjects (22 out of 43) with 2hPG ≥ 200 mg/dL (11.1 mmol/L). In contrast, only 28.6% (26 out of 121) in Mexican Americans, 27.3% (3 out of 11) in other racial/ethnic groups, 25.7% (9 out of 35) in other Hispanics, and 22.5% (50 of 222) in non-Hispanic whites were successfully identified (*P* = 0.004). Based on the difference in HbA1c, FPG, and 2hPG, the HbA1c criterion performed poorly in those with less elevated plasma glucose and less severe diabetes when compared to the 2hPG criterion.

**Figure 2:**
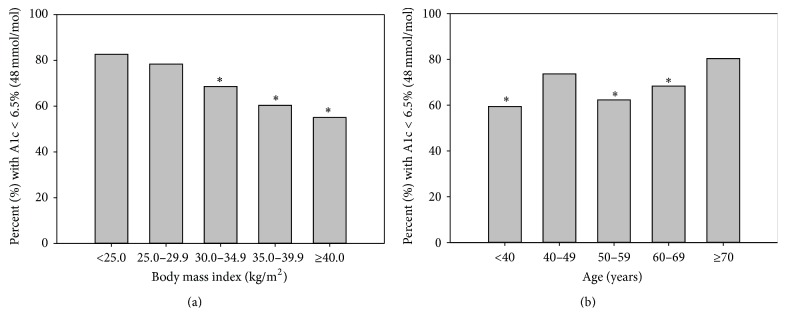
Percent of diabetic subjects by 2-hour plasma glucose criterion with HbA1c < 6.5% (48 mmol/mol) stratified by body mass index (a) and by age group (b). ^*∗*^ *P* < 0.05 when compared to the group with BMI < 25 kg/m^2^ in (a); ^*∗*^ *P* < 0.05 when compared to the group with age ≥ 70 years in (b).

When stratifying the subjects by different BMI (Figure 2(a)), there was a progressive trend of subjects to have an HbA1c < 6.5% (48 mmol/mol) as BMI decreased. In lean subjects (BMI < 25.0 kg/m^2^), only 17.4% of the diabetic subjects defined by 2hPG had an HbA1c ≥ 6.5% (48 mmol/mol). Subjects with higher BMI tended to have fewer missed diagnosis of diabetes when using the HbA1c criterion. The cohort was also stratified into different age groups (Figure 2(b)). In the elderly subjects (age ≥ 70 years), only 19.62% of the diabetic subjects defined by 2hPG had HbA1c ≥ 6.5% (48 mmol/mol). Younger subjects tended to have fewer missed diagnosis of diabetes when using the HbA1c criterion except for the 40–49 age group.

### 3.4. Regression Analysis

**Figure 3:**
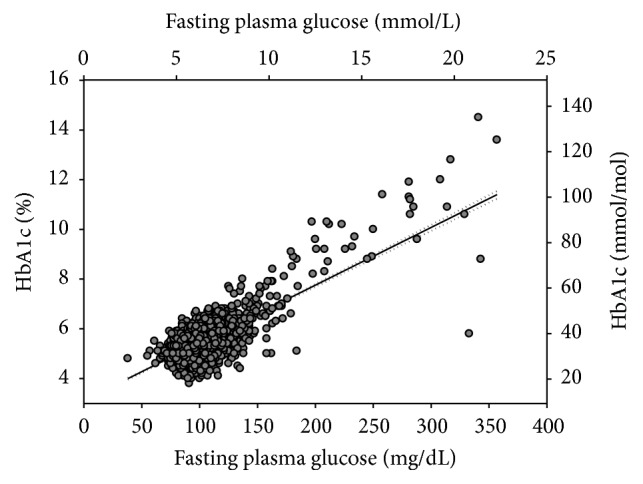
Correlation of HbA1c with fasting plasma glucose. Solid line represents the regression line. Dotted lines represent 95% confidence interval. HbA1c (%) = 3.1151 + 0.0232 × FPG (mg/dL).

**Figure 4:**
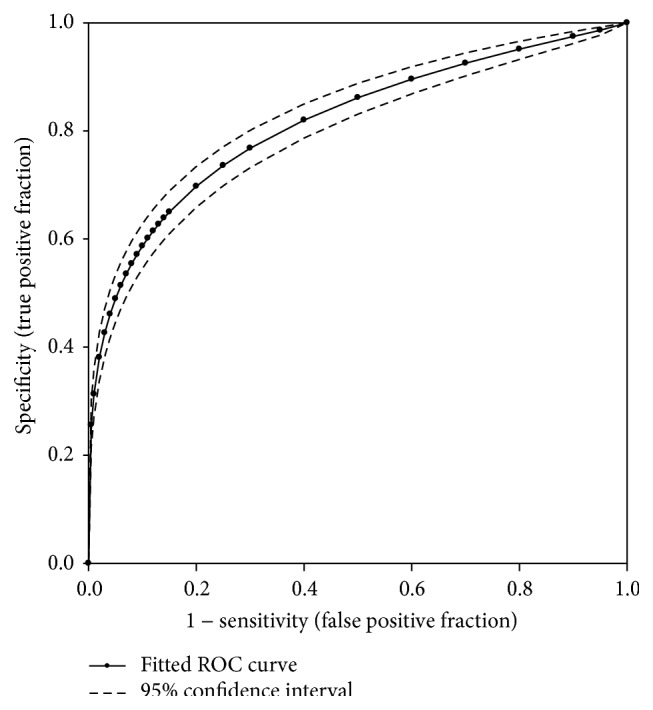
The fitted receiver operating characteristic curve of HbA1c against FPG. Solid line represents the fitted ROC. Dotted lines represent 95% confidence interval.

Regression analysis was used to find an equivalent HbA1c value in respect to FPG (Figure 3). The FPG and HbA1c correlated very well to a linear relationship defined as HbA1c (%) = 3.1151 +0.0232 × FPG (mg/dL) (*r* = 0.7058, *P* < 0.000001). Based on this relationship, a FPG of 126 mg/dL (7.0 mmol/L) correlated closer to an HbA1c of 6.0% (42 mmol/mol). The ROC curve for HbA1c was calculated, as diagnosed by FPG ≥ 126 mg/dL (7.0 mmol/L) (Figure 4). The fitted ROC area was 0.871 (estimated std. error = 0.014). Cohen's kappa coefficient was 0.527 (95% CI: 0.466, 0.588), consistent with fair agreement.

**Figure 5:**
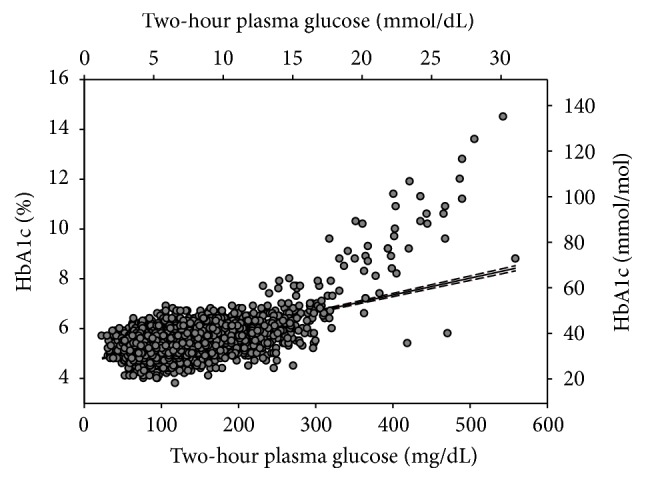
Correlation of HbA1c with 2-hour plasma glucose. Solid line represents the regression line. Dotted lines represent 95% confidence intervals of the regressive line. HbA1c (%) = 4.6500 + 0.0067 × 2hPG (mg/dL).

**Figure 6:**
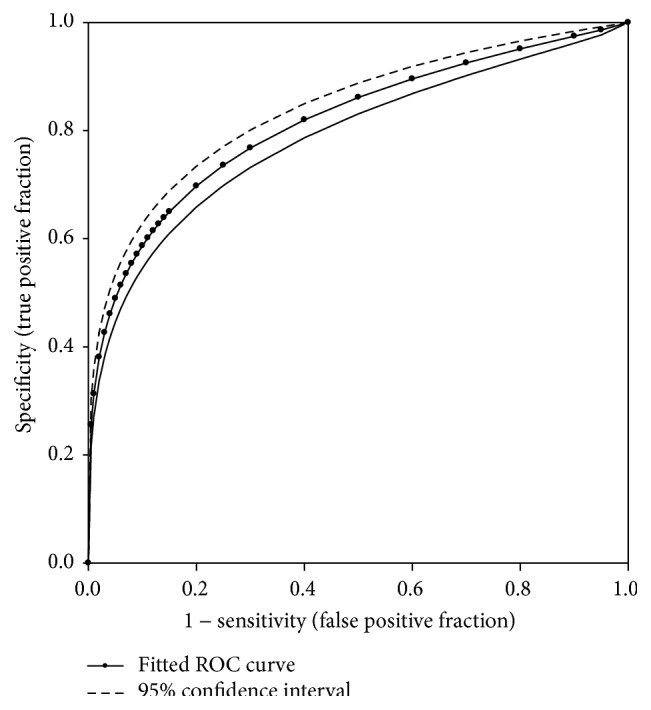
The fitted receiver operating characteristic curve of HbA1c against 2-hour plasma glucose.

Because the cut-off value of HbA1c 6.5% (48 mmol/mL) performed poorly compared to the cut-off value of 2hPG 200 mg/dL (11.1 mmol/L), we examined the correlation between HbA1c and 2hPG (Figure 5) and noted an excellent correlation with a correlation coefficient of 0.5959 (*P* < 0.000001). However, the correlation was heavily weighted by the subjects with 2hPG < 300 mg/dL because they accounted for more than 99% of samples. The estimated regression equation is HbA1c (%) = 4.6500 + 0.0067 × 2hPG (mg/dL). Using this equation, an HbA1c level of 6.0% (42 mmol/mol) corresponds to a 2hPG level of 200 mg/dL (11.1 mmol/L). To further evaluate the role of HbA1c in the diagnosis of diabetes in comparison to 2hPG, the fitted ROC was constructed based on the 5,764 total cases (392 positive cases and 5,372 negative cases) obtained from this study (Figure 6), allowing for an examination of the sensitivity and specificity for the HbA1c criterion. The area under the fitted ROC curve was 0.8159 with an estimated standard error of 0.0128. The accuracy of the HbA1c criterion returned in an adequate range of 0.8 to 0.9 [23]. The agreement between the HbA1c and 2hPG criteria was poor based on Cohen's kappa coefficient of 0.386 (95% CI: 0.334 to 0.439).

## 4. Discussion

We conducted the present study to explore the agreement between plasma glucose (either FPG or 2hPG) and HbA1c in diagnosis of diabetes and to measure the accuracy of using HbA1c ≥ 6.5% (48 mmol/mol) as a diagnostic criterion for diabetes mellitus. We obtained our data from NHANES 2005–2010, which was designed to reflect the noninstitutionalized US population. We found that, of the 245 subjects that had FPG ≥ 126 mg/dL (7.0 mmol/L), only 106 subjects (43.3%) had HbA1c ≥ 6.5% (48 mmol/mol). Out of 392 subjects who had 2hPG ≥ 200 mg/dL (11.1 mmol/L), only 110 subjects (28.1%) had HbA1c ≥ 6.5% (48 mmol/mol). The low sensitivity of the HbA1c criterion in diagnosing diabetes strongly suggests that using an HbA1c ≥ 6.5% (48 mmol/mol) as a criterion for diagnosing diabetes will likely lead to a substantial number of missed diagnoses. These results further underscore the short comings of using the current HbA1c criterion to diagnose diabetes.

In recent years, HbA1c level has been included as a criterion for diagnosis of diabetes. Previously, HbA1c was used to monitor glycemic control in diabetic patients, because it reflects average blood glucose levels over a 2- to 3-month period of time. The diagnostic threshold of HbA1c ≥ 6.5% (48 mmol/mol) was based on the inflection point for the prevalence of retinopathy observed in extensive epidemiological data [10]. However, some studies have shown that there is poor concordance between HbA1c and FPG or 2hPG during an OGTT [24, 25] which are the most widely accepted glucose-based methods for diagnosing diabetes. The Rancho Bernardo study, a cross-sectional study of 2,107 adults without known history of diabetes, showed that the sensitivity and specificity of HbA1c ≥ 6.5% (48 mmol/mol) against OGTT were only 44% and 79%, respectively [24, 25]. Fajans et al. compared HbA1c with FPG in 147 subjects and found that one-third of subjects with early diabetes and impaired glucose tolerance (IGT) had HbA1c < 5.7% (39 mmol/mol) [24, 25]. Prior analyses of NHANES 2003–2006 data showed that the prevalence of undiagnosed diabetes using the HbA1c ≥ 6.5% (48 mmol/mol) criterion was only one-third of that using the FPG ≥ 126 mg/dL (7.0 mmol/L) criterion [26]. Using the largest samples in the reported studies to date, we confirmed the low sensitivity of HbA1c criterion in comparison to FPG criterion. Nonetheless, HbA1c remains a recommended diagnostic tool because of its practicality and convenience based on the expert opinion in cross-sectional observation studies [10]. Consequently, the actual number of individuals diagnosed with diabetes may increase due to its frequent use. Other benefits of HbA1c over FPG include stronger correlation with retinopathy [27] and less variability in day-to-day within-person variance (<2% for HbA1c versus 12–15% for FPG) [28].

Although using HbA1C ≥ 6.5% (48 mmol/mol) was advocated for diagnosis of diabetes, few studies have compared the sensitivity of HbA1c with 2hPG, and one study noted that 2hPG actually performed better than HbA1c in classifying diabetes [29] which was in respect to the cardiovascular complications but not retinopathy. Among Asian Americans and Native Hawaiians, the sensitivity of HbA1c ≥ 6.5% (48 mmol/mol) to define diabetes was 40.0% by 2hPG and 68.9% by FPG only [30]. However, 64.8% of Asian subjects with diabetes had isolated postchallenge hyperglycemia, and the sensitivity of HbA1c ≥ 6.5% (48 mmol/mol) to define isolated 2hPG was only 19.1% [30]. In a population undergoing coronary angiography in Taiwan [31], HbA1c ≥ 6.5% (48 mmol/mol) was noted in only 39.2% of patients with 2hPG ≥ 200 mg/dL (11.1 mmol/L). Compared to these three studies, our study included the largest sample set and confirmed the low sensitivity of HbA1c criterion. Several small studies showed a low sensitivity of HbA1c in comparison with the results of OGTT [25, 32–36]. However, no information was provided in these studies specifically in reference to 2hPG. Nevertheless, their observations are consistent with our results that an HbA1c ≥ 6.5% (48 mmol/mol) has a low sensitivity in diagnosing diabetes.

Our study showed that, in reference to FPG, the current HbA1c criterion had a low sensitivity (43.3%) and may be inadequate to detect individuals with diabetes mellitus. Consequently, we suggest that the HbA1c cut-off value should be revised for better sensitivity to better identify individuals in an early diabetic state. Correctly identifying the early diabetic state could prevent micro- and macrovascular complications or delay progression. Based on our regression analysis, the equivalent HbA1c value in respect to FPG 126 mg/dL (7.0 mmol/L) was closer to 6.0% (42 mmol/mol). From our current sample set, if we use the cut-off value of HbA1c ≥ 6.0% (42 mmol/mol), the sensitivity and specificity are 69.8% and 91.9%, respectively. Our findings are similar to those of a meta-analysis [37]. Bennett et al. reported that HbA1c of 6.1% (43 mmol/mol) was the recommended optimum cut-off point based on 9 studies whose reported sensitivity and specificity ranged from 78 to 81% and from 79 to 84%, respectively [37]. More recent studies have shown similar findings [25, 38].

In this study, the regression analysis demonstrated good correlation between FPG and HbA1c (*r* = 0.7058, *P* < 0.000001). To assess the diagnostic accuracy of HbA1c, the fitted ROC area was calculated to be 0.871 (estimated std. error = 0.014) which is in agreement with another study that compared HbA1c, FPG, and 2hPG in adult Italian Caucasians [39]. Our findings demonstrated that HbA1c is a relatively good diagnostic test when compared with FPG. Nonetheless, we suggest that the cut-off value of HbA1c for diagnosing diabetes should be lowered to HbA1c ≥ 6.0% (42 mmol/mol) which is more equivalent to FPG ≥ 126 mg/dL (7.0 mmol/L).

One of the advantages of using an HbA1c test is that it measures average blood glucose over a 3-month period. HbA1c also does not require patients to fast unlike FPG and is performed via a simple venipuncture unlike 2hPG, which entails the patient to ingest 75 grams of oral glucose between blood draws. Because patients' fasting status does not need to be verified and the cumbersome procedure of coordinating the ingestion of oral glucose and laboratory draws are not necessary, testing for HbA1c provides convenience for patients and simplifies diabetes screening for health providers. However, limitations of HbA1c in reflecting chronic hyperglycemia have been reported [40]. In patients with high red blood cell turnover, HbA1c may be falsely lowered because the shortened life spans of red blood cells may lower the percentage of glycated hemoglobin regardless of the level of hyperglycemia in the blood. Patients with hemoglobinopathy will also have unreliable HbA1c. Differing levels of glycation, which have been reported in certain ethnic/racial groups, may also contribute to the discordance between HbA1c and glucose levels in patient's serum [41].

The diagnosis of diabetes is not established or confirmed based on a single test but rather by repeated measurement of FPG, 2hPG, or HbA1c [22]. Given the low sensitivity of HbA1c ≥ 6.5% (48 mmol/mol) in diagnosing diabetes in this study, we do not recommend using this criterion as a screening tool because it tends to miss the majority of the cases when compared to FPG and 2hPG. However, HbA1c had lower within-person variability (within-person coefficient of variation (CV): 3.6%; 95% CI: 3.2, 4.0) as compared to 2hPG (CV: 16.7%; 95% CI: 15.0, 18.3) and FPG (CV: 5.7%; 95% CI: 5.3, 6.1) [42]. Thus, HbA1c could be more reproducible than 2hPG and FGP.

Considerable strengths of the study include the large sample size, the ethnically diverse population, and the wide range of ages in the cohort. The study cohort included 245 and 392 patients who were previously undiagnosed with diabetes using FPG criterion and 2hPG criterion, respectively. By analyzing a large, diverse population and limiting analysis to patients who never had a previous diagnosis of diabetes, our study is more representative of the population typically screened for diabetes in clinical practice.

A clear limitation of the study is that although it was clearly demonstrated that the current HbA1c criterion failed to detect a substantial proportion of patients that have diabetes using FPG or 2hPG levels, the current study did not allow for any assessment of the clinical significance of this failure. Further studies should focus on whether diabetes related complications, such as neuropathy and nephropathy, increase when HbA1c reaches 6.0% (42 mmol/mol). Future studies should also investigate the temporal influences on the discordance between the HbA1c and FPG/2hPG criteria. Studying this discordance is especially clinically relevant because patients with missed diagnosis using HbA1c may eventually be diagnosed with diabetes in the next few months or years using the same HbA1C criterion. Whether this delay in diagnosis will have deleterious effect on the health of an individual should also be investigated.

As summarized in Table 6, to accurately define the prevalence of diabetes and to avoid underdiagnosis of diabetes, HbA1c should be used cautiously and as a supplement to FPG and 2hPG. Our data demonstrate (Tables 3 and 5) that the HbA1c criterion is much less sensitive than FPG and 2hPG in diagnosing diabetes in those with mild disease. To be in agreement with FPG and 2hPG, HbA1c of 6.0% (42 mmol/mol) could be used as the cut-off value to prevent delaying diagnosis, surveillance, and ultimately treatment of diabetes in patients. However, before recommendations of using this cut-off value, a longitudinal study is required to demonstrate whether it truly affects the long-term diabetic complications. Regardless, our results support that when the diagnosis of diabetes by HbA1c is in doubt, FPG and/or 2hPG should be used for early diagnosis of diabetes.

## Figures and Tables

**Table 1:** Clinical features of studied subjects.

	Mean (*n*)		STD^*∗*^ (%)
*n*	5,764		
Age, year	46	±	19
Gender, female	2,873		49.8%
Body mass index, kg/m^2^	28.4	±	6.5
Systolic blood pressure, mmHg	121	±	17
Diastolic blood pressure, mmHg	68	±	12

Current smoking, yes	1,479		25.7%
Alcohol consumption, yes	3,801		65.9%
Family history of diabetes, yes	1,983		34.4%

HbA1c, %	5.5	±	0.6
HbA1c, mmol/mol	36	±	6
Fasting plasma glucose, mg/dL	101	±	18
Fasting plasma glucose, mmol/L	6.1	±	1.0
Two-hour plasma glucose, mg/dL	119	±	52
Two-hour plasma glucose, mmol/L	6.6	±	2.9

Racial/ethnic group			
Mexican Americans	1,117		19.4%
Other Hispanics	519		9.0%
Non-Hispanic Whites	2,812		48.9%
Non-Hispanic Blacks	1,060		18.4%
Others	250		4.3%

^*∗*^STD: standard deviation.

**Table 2:** Percentage of subjects meeting diagnostic criteria for diabetes by fasting plasma glucose and hemoglobin A1c.

		Fasting plasma glucose				Subtotal
		<126 mg/dL (<7 mmol/L)		≥126 mg/dL (≥7 mmol/L)		
HbA1c	<6.5% (<48 mmol/mol)	5,479	99.3%	139	56.7%	5,618
	≥6.5% (<48 mmol/mol)	40	0.7%	106	43.3%	146

Subtotal		5,519		245		5,764

*n* with column percent.

**Table 3:** Comparison of clinical characteristics of subjects with fasting plasma glucose ≥126 mg/dL (≥7.0 mmol/L) stratified by HbA1c < or ≥ 6.5% (48 mmol/mol).

	Diabetes by fasting glucose		*P*
	HbA1c < 6.5% (<48 mmol/mol)	HbA1c ≥ 6.5% (≥48 mmol/mol)	
*n*	139	106	
Age, year	61 ± 15	59 ± 15	NS
Gender, female	49 (35.3%)	39 (36.8%)	NS
Body mass index, kg/m^2^	31.4 ± 7.2	33.0 ± 7.0	NS
Systolic blood pressure, mmHg	129 ± 20	132 ± 20	NS
Diastolic blood pressure, mmHg	71 ± 13	71 ± 15	NS

Current smoking, yes	33 (23.7%)	34 (32.1%)	NS
Alcohol consumption, yes	98 (70.5%)	69 (65.1%)	NS
Family history of diabetes, yes	61 (43.9%)	33 (31.1%)	NS

HbA1c, %	5.8 ± 0.5	8.0 ± 1.8	<0.0001
HbA1c, mmol/mol	40 ± 3	64 ± 14	
Fasting plasma glucose, mg/dL	136 ± 19	180 ± 58	<0.0001
Fasting plasma glucose, mmol/L	7.6 ± 1.1	10.0 ± 3.2	
Two-hour plasma glucose, mg/dL	195 ± 64	315 ± 90	<0.0001
Two-hour plasma glucose, mmol/L	10.8 ± 3.6	17.5 ± 5.0	

Racial/ethnic group			NS
Mexican Americans	26 (18.7%)	26 (24.5%)	
Other Hispanics	15 (10.8%)	9 (8.5%)	
Non-Hispanic Whites	79 (56.8%)	48 (45.3%)	
Non-Hispanic Blacks	18 (13.0%)	19 (17.9%)	
Others	1 (0.7%)	4 (3.8%)	

Mean ± standard deviation or *n* with percent; NS, not significant.

**Table 4:** Distribution of diabetic states by 2-hour plasma glucose and HbA1c.

		Two-hour plasma glucose				Subtotal
		<200 mg/dL (<11.1 mmol/L)		≥200 mg/dL (≥11.1 mmol/L)		
HbA1c	<6.5% (<48 mmol/mol)	5,336	99.3%	282	71.9%	5,618
	≥6.5% (≥48 mmol/mol)	36	0.7%	110	28.1%	146

Subtotal		5,372		392		5,764

*n* with column percent.

**Table 5:** Comparison of clinical characteristics of subjects with 2-hour plasma glucose ≥200 mg/dL (≥11.1 mmol/L) stratified by HbA1c < or ≥ 6.5% (48 mmol/mol).

	Diabetes by 2-hour plasma glucose		*P*
	HbA1c < 6.5% (<48 mmol/mol)	HbA1c ≥ 6.5% (≥48 mmol/mol)	
*n*	282	110	
Age, year	64 ± 15	60 ± 15	0.01
Gender, female	150 (53.2%)	42 (38.2%)	0.008
Body mass index, kg/m^2^	29.7 ± 6.1	33.0 ± 6.6	<0.0001
Systolic blood pressure, mmHg	132 ± 20	132 ± 20	NS
Diastolic blood pressure, mmHg	68 ± 13	70 ± 14	NS

Current smoking, yes	51 (18.1%)	32 (29.1%)	0.02
Alcohol consumption, yes	192 (68.1%)	74 (67.3%)	NS
Family history of diabetes, yes	110 (39.0%)	52 (47.3%)	NS

HbA1c, %	5.7 ± 0.4	7.9 ± 1.8	<0.0001
HbA1c, mmol/mol	39 ± 5	63 ± 18	
Fasting plasma glucose, mg/dL	83 ± 20	176 ± 60	<0.0001
Fasting plasma glucose, mmol/L	6.5 ± 1.1	9.8 ± 3.3	
Two-hour plasma glucose, mg/dL	232 ± 31	318 ± 82	<0.0001
Two-hour plasma glucose, mmol/L	12.9 ± 1.8	17.7 ± 4.6	

Racial/ethnic group			0.004
Mexican Americans	95 (19.5%)	26 (23.6%)	
Other Hispanics	26 (9.2%)	9 (8.2%)	
Non-Hispanic Whites	172 (61.0%)	50 (45.5%)	
Non-Hispanic Blacks	21 (7.5%)	22 (20.0%)	
Others	8 (2.8%)	3 (2.7%)	

Mean ± standard deviation or *n* with percent; NS, not significant.

**Table 6:** Performance of HbA1c on the diagnosis of diabetes in reference to fasting and 2-hour plasma glucose.

	Fasting plasma glucose	Two-hour plasma glucose
Sensitivity	43.3%	28.1%
Specificity	99.3%	99.3%
Positive predictive value	72.6%	75.3%
Negative predictive value	97.5%	95.0%
False positive rate	0.7%	0.7%
False negative rate	56.7%	71.9%
ROC area under curve (95% CI)	0.871 (0.842, 0.899)	0.816 (0.791, 0.841)
Cohen's kappa coefficient (95% CI)	0.527 (0.467, 0.588)	0.386 (0.334, 0.439)

## Abbreviations

2hPG: Two-hour plasma glucose
BMI: Body mass index
DM: Diabetes mellitus
FPG: Fasting plasma glucose
NHANES: National Health and Nutrition Examination Survey
OGTT: Oral glucose tolerance test
ROC: Receiver operating characteristic curve.
